# Electrosurgical Clip Laceration to Facilitate Transcatheter Tricuspid Valve Replacement After Suboptimal Edge-To-Edge Repair

**DOI:** 10.1016/j.shj.2026.101103

**Published:** 2026-07-23

**Authors:** Fabio Solis-Jiménez, Daniel Arnaldo Pinto-Torres, Pilar Jiménez-Quevedo, Patricia Mahia-Casado, Eduardo Pozo-Osinalde, Luis Nombela-Franco

**Affiliations:** Cardiovascular Institute, Hospital Clínico San Carlos, Instituto de Investigación Sanitaria del Hospital Clínico San Carlos (IdISSC), Madrid, Spain

**Keywords:** Electrosurgical clip laceration, Electrosurgical Laceration and Stabilization of Clip devices (ELASTA), Failed edge-to-edge repair, Transcatheter tricuspid valve replacement (TTVR), Tricuspid regurgitation (TR), Valve-in-clip

## Abstract

• Transcatheter tricuspid valve replacement after prior failing tricuspid transcatheter edge-to-edge repair is feasible.
• Clip laceration modifies anatomy to create a clear landing zone.
• The role of imaging guidance is highlighted step by step.

Despite the established efficacy of tricuspid transcatheter edge-to-edge repair (T-TEER) for tricuspid regurgitation (TR), some patients experience suboptimal results with significant residual TR immediately after the procedure or at follow-up. Although transcatheter tricuspid valve replacement (TTVR) is an emerging solution, in these cases, the presence of well-attached central clips can preclude valve expansion and anchoring. This report describes a percutaneous strategy that adapts the electrosurgical laceration technique from the mitral to the tricuspid position to facilitate successful TTVR.^1^

An 81-year-old male with massive TR underwent T-TEER with implantation of 3 devices (2 anteroseptal and 1 posteroseptal). Despite an initially good procedural and echocardiographic result, severe TR recurred at 18-month follow-up. TTVR was considered; however, due to concern that the central orifice would be inadequate for a proper valve expansion and possible paravalvular leak between devices, the decision was made to perform a partial laceration of the central clip to facilitate TTVR (Figure 1). Subsequently, TTVR was performed using a 56-mm EVOQUE valve (Edwards Lifesciences, Irvine, California). The space created by the previous steps enabled its adequate implantation, resulting resolution of the TR (Figure 2). The procedural sequence is shown in Video 1.

**Figure 1:**
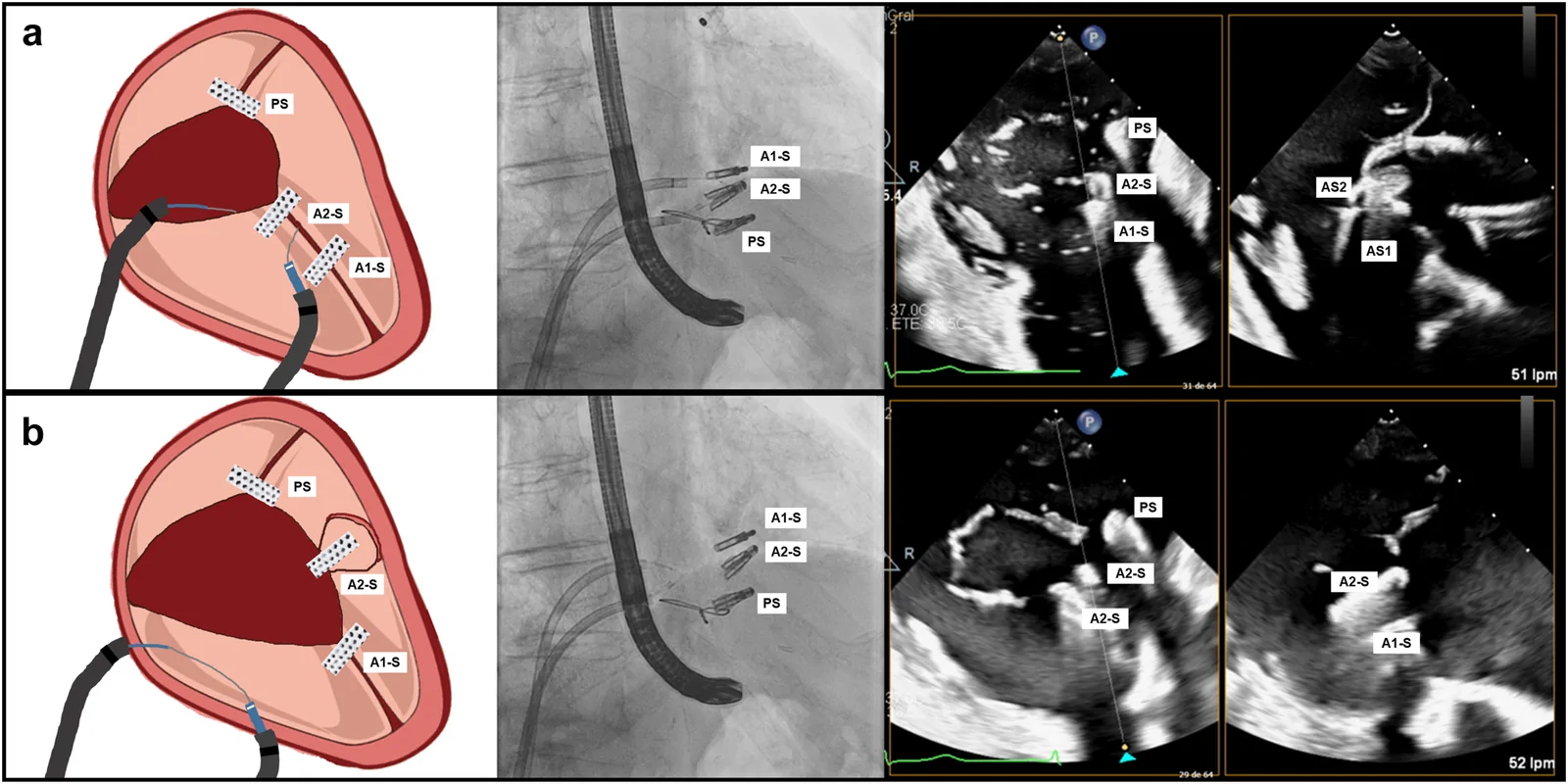
**Clip liberation to facilitate TTVR.** The system used to perform laceration consisted of 2 8.5-Fr Agilis NxT steerable sheaths, with a microcatheter positioned within 1 sheath and a 6-Fr Judkins right guide catheter within the other. After externalization of a 300-cm long ASTATO coronary wire across the main orifice and between the A1-S and A2-S clips (a), the wire was electrified (energy 60 W) while both Agilis catheters were gently retracted to lacerate the anterior leaflet attached to the A2-S clip (b). Abbreviations: PS, posteroseptal; TTVR, transcatheter tricuspid valve replacement.

**Figure 2:**
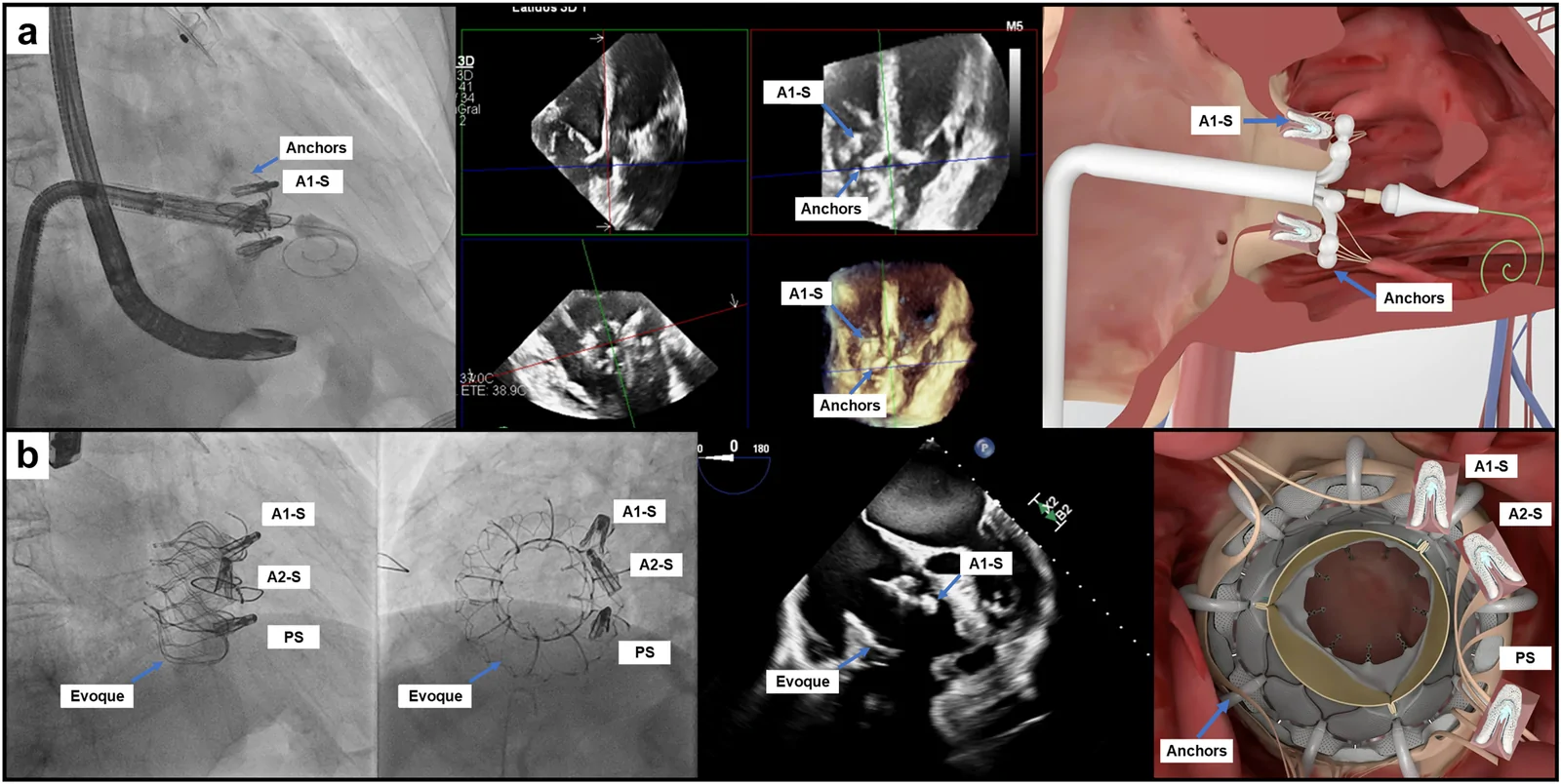
**Procedural imaging and schematic representation of Evoque TTVR after clip liberation.** (a) Previous implanted T-TEER devices being captured by leaflets anchors of TTVR on fluoroscopy and transesophageal echocardiography, with corresponding schematic representation. (b) Final Evoque valve position and interaction with previous T-TEER devices. Abbreviations: PS, posteroseptal; T-TEER, tricuspid transcatheter edge-to-edge repair; TTVR, transcatheter tricuspid valve replacement.

This case demonstrates the feasibility of a percutaneous, 2-step strategy for managing failed T-TEER. Targeted electrosurgical laceration of a central, well-attached clip can safely modify the anatomy to facilitate subsequent TTVR. Procedural planning is critical to the success of the intervention, with 2 key considerations. First, careful selection of the clip and leaflet to be lacerated is essential. To create a large central orifice for TTVR device implantation, the target clip should be the most centrally positioned and farthest from the commissures. Typically, when the distance from the clip to the annulus is less than 10–15 mm, this technique may not be necessary, as the prosthesis can displace the clip toward the commissure. The preferred leaflet for laceration is the nonseptal leaflet, as it is generally longer than the septal leaflet and more easily captured by the TTVR device, even after laceration. Second, valve sizing should be based on the native annular dimensions when leaflet laceration is planned.^2^ This approach represents an important advancement in the interventional lifetime management of tricuspid valve disease and appears to be both safe and effective in this patient population. Further experience is needed to standardize patient selection criteria and optimize laceration strategies.

## Ethics Statement

This manuscript was carried out in accordance with the appropriate ethical guidelines.

## Funding

The authors have no funding to report.

## Disclosure Statement

Luis Nombela-Franco reports a relationship with Edwards Lifesciences Corp that includes consulting or advisory and speaking and lecture fees, and he reports a relationship with Abbott Vascular Inc that includes consulting or advisory and speaking and lecture fees. Given his role as an associate editor of *Structural Heart*, he had no involvement in the peer review of this article and had no access to information regarding its peer review. Full responsibility for the editorial process for this article was delegated to another journal editor. Eduardo Pozo-Osinalde, Pilar Jimenez-Quevedo, and Luis Nombela-Franco are proctor for Abbott Vascular and Edwards Lifesciences.

The other authors had no conflicts to declare.
